# Evidence for crustal brines and deep fluid infiltration in an oceanic transform fault

**DOI:** 10.1126/sciadv.adu3661

**Published:** 2025-04-11

**Authors:** Christine Chesley, Rob Evans, Jessica M. Warren, Andrew C. Gase, Jacob Perez, Christopher Armerding, Hannah Brewer, Paige Koenig, Eric Attias, Bailey L. Fluegel, Jae-Deok Kim, Natalie Hummel, Katherine Enright, Emilia Topp-Johnson, Margaret S. Boettcher

**Affiliations:** ^1^ Geology and Geophysics Department, Woods Hole Oceanographic Institution, Woods Hole, MA 02543, USA.; ^2^ Department of Earth Sciences, University of Delaware, Newark, DE 19716, USA.; ^3^ Department of Geoscience, Boise State University, Boise, ID 83725, USA.; ^4^ Cecil H. and Ida Green Institute of Geophysics and Planetary Physics, Scripps Institution of Oceanography, University of California San Diego, La Jolla, CA 92093, USA.; ^5^ Geology Department, Western Washington University, Bellingham, WA 98225, USA.; ^6^ Institute for Geophysics, University of Texas at Austin, Austin, TX 78712, USA.; ^7^ MIT-WHOI Joint Program in Oceanography/Applied Ocean Science and Engineering, Woods Hole, MA 02543, USA.; ^8^ School of Psychology, University of Southern Maine, Portland, OR 04103, USA.; ^9^ Fu Foundation School of Engineering and Applied Science, Columbia University, New York, NY 10027, USA.; ^10^ School of Marine Science and Ocean Engineering, University of New Hampshire, Durham, NH 03824, USA.

## Abstract

Although oceanic transform faults (OTFs) are ubiquitous plate boundaries, the geological processes occurring along these systems remain underexplored. The Gofar OTF of the East Pacific Rise has gained attention due to its predictable, yet enigmatic, earthquake cycle. Here, we present results from the first ever controlled-source electromagnetic survey of an OTF, which sampled Gofar. We find that the fault is characterized by a subvertical conductor, which extends into the lower crust and thus implies deep fluid penetration. We also image subhorizontal crustal conductors distributed asymmetrically about the fault. We interpret these subhorizontal anomalies as crustal brines, and we suggest that the high permeability of the fault combined with the influence of melt in the transform domain can promote hydrothermal circulation and brine condensation at OTFs.

## Teaser

Electrical resistivity images at an oceanic transform fault reveal very salty fluids, which require far-off-axis melt to form.

## INTRODUCTION

Oceanic transform faults (OTFs) connect the global network of mid-ocean ridges (MORs), undergoing predominantly strike-slip motion in response to the spreading of plates. Despite their prevalence throughout the seafloor, compared to MORs and subduction zones, relatively few studies have focused on OTFs and to date no electromagnetic (EM) geophysical surveys have specifically probed an OTF. Fundamental conundrums about these features persist, especially with regard to earthquake dynamics, and their status as conservative plate boundaries, where the lithosphere is neither created nor destroyed, has more recently been contested. For instance, although OTFs can extend for hundreds of kilometers on the seafloor, the largest events they typically produce are *M*_w_ ≤ 7 and only ~20% of their stored seismic moment is released in earthquakes (*1*–*4*). This deficit suggests that OTFs release most of their accumulated stresses aseismically. In addition, analyses of gravity anomalies (*5*), seismic shear wave splitting (*6*), and topography of OTFs (*7*) point to a potential for magmatic processes to be active along intermediate and fast-slipping faults.

The Gofar OTF of the equatorial East Pacific Rise (EPR) has been the site of repeated seismological investigations in part because its fast slip rate (~140 mm/year) results in a short (~5 to 6 year) earthquake recurrence interval (*8*–*10*). The westernmost portion of this fault system ruptures quasiperiodically in *M*_w_ ~ 6 events that occur on two fully coupled asperities separated by a persistent barrier zone, which displays abundant swarms of microseismicity to depths greater than the expected brittle-ductile transition at 600°C (*8*, *10*, *11*). The cause of this barrier zone remains unknown, but wide-angle seismic refraction data (*12*, *13*), local seismicity experiments (*8*, *10*, *14*–*16*), and numerical rate-and-state friction models (*17*) collectively invoke deep and elevated fluid content and/or intensified fault damage as the most plausible mechanism for generating and sustaining the barrier zone. However, these studies, confined mainly to the fault valley, are limited in their spatial extent, and thus largely fail to identify any potential off-fault structures and processes that may influence the dynamics of the OTF and its barrier zone.

**Fig. 1.**
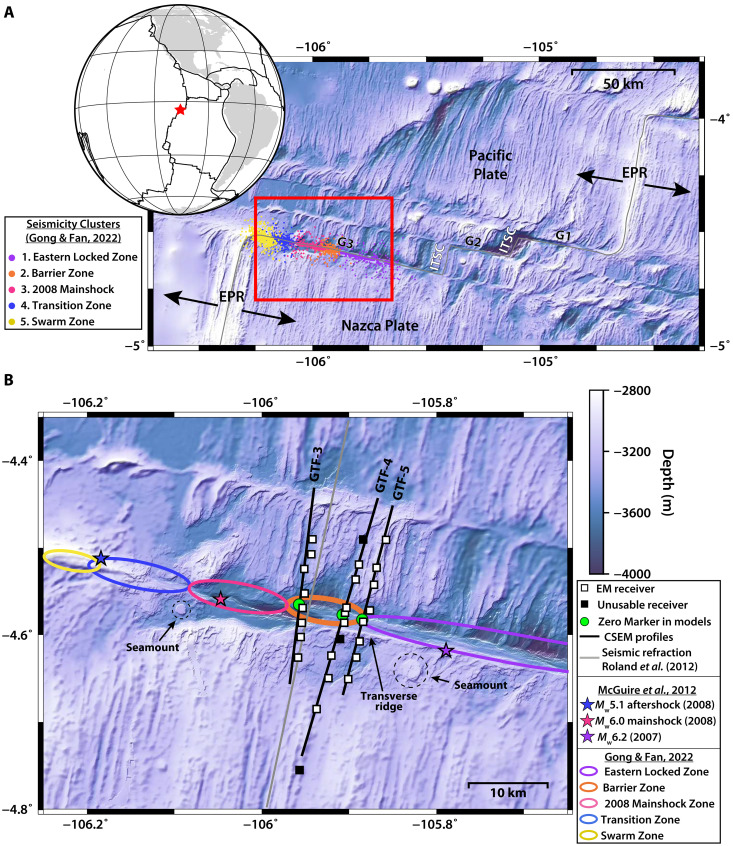
Bathymetric map of survey region. (**A**) The East Pacific Rise (EPR) in the vicinity of the Gofar Transform Fault (red star in the inset globe). The three segments of Gofar are labeled G1, G2, and G3. Intratransform spreading centers are labeled ITSC. Arrows indicate relative plate motion of the Pacific and Nazca Plates. Relocated seismicity from ocean-bottom seismometers deployed in 2008 (*8*) are shown as dots where the colors indicate distinct clusters described in ref. (*10*) (see legend on left). Red square is the region shown in (B). (**B**) Close-up view of study area. White and black squares are ocean-bottom electromagnetometers with usable and unusable data, respectively. Black lines are CSEM profiles. Gray line is the seismic refraction profile of (*12*). *M*_w_ ≥ 5.1 earthquakes in 2007 and 2008 from (*8*) are shown as stars. Ellipses indicate the approximate along-fault extent of seismicity clusters from (*10*), where the orange ellipse is the earthquake rupture barrier zone (see legend on right). Seamounts mentioned in the text are indicated by gray dashed circles. These seamounts preserve a circular shape, which suggests that they formed away from the ridge, although they have not been dated. Bathymetry is from GMRT (*70*).

To better characterize the geophysical properties of OTFs in general and the Gofar barrier zone in particular, we collected controlled-source EM (CSEM) data along the western end of the Gofar OTF in January to February 2022. We deployed 27 ocean bottom EM receivers in three ~30-km–long, fault-perpendicular profiles (Fig. 1). The two westernmost profiles intersected the barrier zone whereas the third straddled the seismically defined transition between the barrier zone and a rupture asperity to the east (*8*, *10*). Here, we present the first electrical resistivity models of an OTF obtained using CSEM. We constrain resistivity (conductivity^−1^) in the shallow seafloor (≤10 km below seafloor) of the Gofar OTF. Because CSEM data are exceptionally sensitive to the interconnection of conductive phases (e.g., seawater, melt, and metals) within relatively resistive crustal rocks (*18*–*21*), these electrical resistivity models are invaluable in our efforts to map fluid availability and composition at the Gofar OTF.

## RESULTS

### Asymmetrical electrical resistivity across the Gofar OTF

**Fig. 2.**
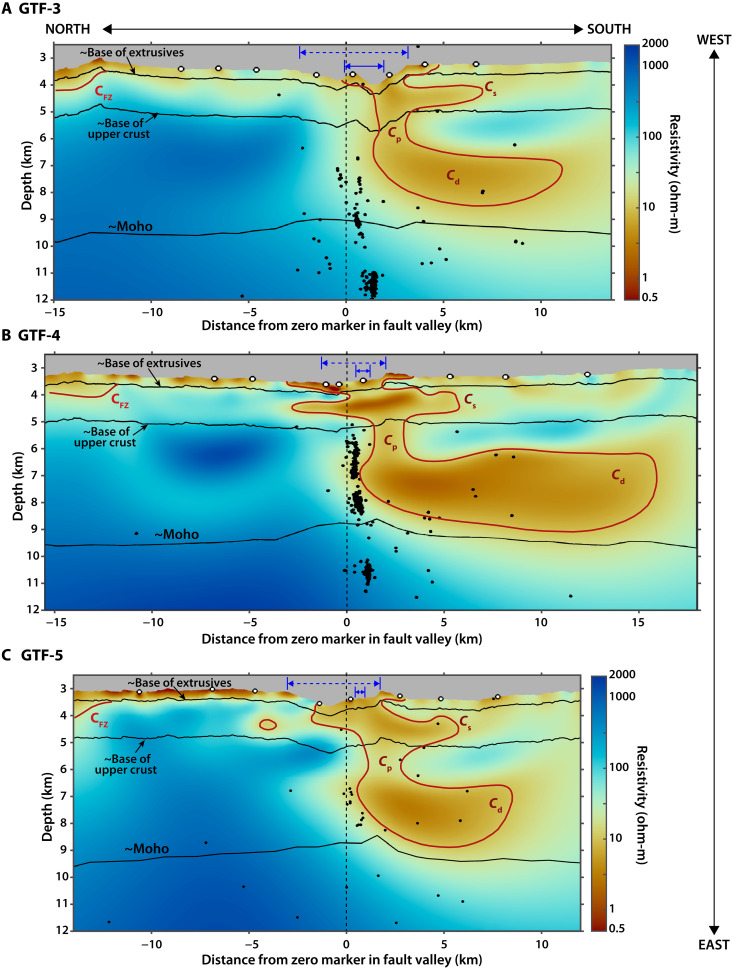
Resistivity model results. (**A**) to (**C**) show the final resistivity model for profiles GTF-3, GTF-4, and GTF-5, respectively. The vertical axis is depth below sea level and an approximate center point in the fault is denoted by a vertical, black dashed line (shown as green circles in Fig. 1). Note that we do not know the exact location of the fault center and that the fault damage zone likely varies in width (*71*). Conductivity anomalies *C*_p_, *C*_s_, *C*_d_, and *C*_FZ_, which are discussed in the text, are outlined in red here. Approximate layer boundaries are shown as solid black lines. The base of the extrusives and base of the upper crust are estimated from the resistivity models (see text). The Moho boundary is approximated from the nearby seismic tomography profile (*12*) by adding this depth below seafloor to each profile’s topography. Seismicity in Fig. 1A within 250 m of each profile is shown as black dots (*10*). The approximate width of the damage zone, estimated from high-resolution seafloor bathymetry data in (*71*), is shown by solid blue lines and bars at the top of each profile. Our estimates of the transform valley width based on the bathymetry in Fig. 1B are shown as dashed blue lines and bars.

We modeled the CSEM data for two-dimensional (2D) isotropic electrical resistivity (conductivity^−1^) structure using nonlinear, regularized inversion (*22*) (Materials and Methods). Anisotropic inversions were also performed, and they suggest that anisotropy is not required to fit the data (figs. S1 and S2). The resistivity model for each profile is broadly similar (Fig. 2).

To the north of the Gofar OTF, the resistivity of the marginally older [~0.7 to 0.9 million years (Ma)] Pacific Plate is typical of normal, intraplate oceanic crust (fig. S3) in which resistivity increases monotonically with depth due predominantly to closure of pore spaces (*18*–*21*, *23*). Exceptions to this are most apparent in profile GTF-4 where a low resistivity, arcuate feature appears in the lower crust (Fig. 2B). This low-resistivity zone is less pronounced in GTF-3. Sensitivity analyses indicate this low-resistivity zone could be an inversion artefact, though more data are needed to test this further (fig. S4; Materials and Methods). In each preferred resistivity model north of the OTF, a thin layer of conductive (0.5 to 20 ohm-m) material overlies a slightly more resistive (20 to 200 ohm-m) layer in which resistivity increases rapidly. These are typical ranges for the extrusive and sheeted dike sections, respectively, of normal oceanic crust (fig. S3) (*18*–*21*, *24*). Thus, to estimate the base of the extrusive and sheeted dike sections, we averaged the depth to the 20 ohm-m (~355 m below seafloor) and 200 ohm-m (~1735 mbsf) contours, respectively, across all three profiles at lateral distances of −9 to −6 km along the horizontal axis, where the resistivity structure appears normal for oceanic crust. The thicknesses of each layer lie within the range for young, fast-spreading-derived oceanic crust determined from seismic profiling (*25*, *26*). Below these upper crustal layers is the more resistive lower crust (200 to 1000 ohm-m). A previous seismic refraction experiment at Gofar (*12*) gives the approximate depth to the Moho in this region, which is shown in Fig. 2.

South of the Gofar transform valley, the CSEM data reveal a distinct resistivity structure for the slightly younger (~0.5 to 0.7 Ma) Nazca Plate that starkly contrasts the resistivity model to the north, even after accounting for age-based temperature differences and thermal upwelling within OTFs (*11*, *27*). In other words, the resistivity structure south of the Gofar OTF is not simply a more conductive version of its northern counterpart, which would be expected if thermal (“conductive”) cooling were the only mechanism responsible for generating variations in the electrical properties across an OTF.

**Fig. 3.**
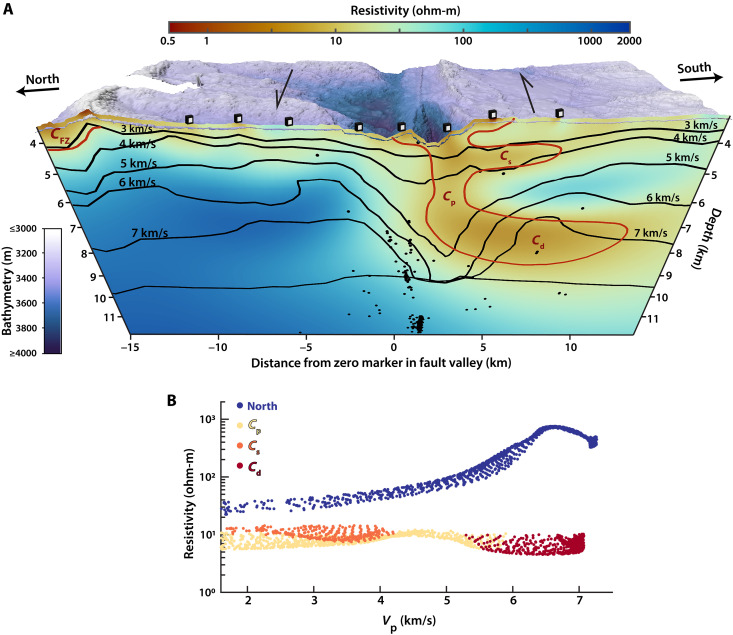
Comparison of resistivity and P-wave velocity at Gofar. (**A**) Resistivity model from GTF-3 with *V*_P_ contours from (*12*) overlain in black. Conductivity anomalies are outlined in red and discussed in the text. Seismicity from (*10*) within 250 m laterally of the profile is shown as black dots. Multibeam bathymetry shown was collected during this research cruise. (**B**) Cross-plots of resistivity and velocity for the northern part of the fault between −9 to −6 km and conductors *C*_p_, *C*_s_, and *C*_d_ are shown in dark blue, yellow, orange, and red, respectively. The *V*_P_-resistivity relationship shows a positively correlated trend north of the fault except where the low-resistivity zone occurs at the base of the crust (*V*_P_ > 6.5 km/s). In contrast, *V*_P_ and resistivity are nearly uncorrelated (coefficient of determination < 0.1) for each conductor south of the fault, although *C*_p_ and *C*_s_ occupy regions of low *V*_P_.

Each of our resistivity models instead reveals conspicuous conductivity anomalies to the south of the OTF in both the upper and lower crust (Fig. 2). In the purported sheeted dike section of the crust, a shallow, subhorizontal conductor (*C*_s_; 1.5 to 15 ohm-m) extends roughly 7 km south of the fault and up to 4.5 km north of the fault. A pipe-like conductor (*C*_p_; 5 to 20 ohm-m) connects this shallow anomaly to a highly conductive (2 to 10 ohm-m) lower crustal body (*C*_d_) that extends to the south of the fault. This resistivity structure is particularly remarkable because, to date, such subhorizontal crustal conductors have not been observed in 2D CSEM surveys of oceanic crust at MORs (*20*), subduction zones (*19*, *21*), or on the inactive abyssal plain (*19*) and may indicate that they are unique features of OTFs, perhaps fast-slipping ones in particular. Because of the high conductivity of *C*_d_, we cannot ascertain whether *C*_d_ is confined to the lower crust or extends to mantle depths given the transmission frequency and geometry of the survey (fig. S5; Materials Methods). Microseismicity detected using ocean bottom seismometers from a 2008 deployment (*8*, *10*) appears to lie mostly north of *C*_p_ and *C*_d_ (Figs. 2 and 3), which is particularly noticeable in profile GTF-4.

*C*_p_ is not centered directly beneath the fault valley, where seismicity presumptively delineates the fault trace (*10*), but rather, this anomaly is offset toward the south of the fault and bears some similarity in shape to the low–P-wave (*V*_P_) velocity zone observed in (*12*), which is also offset south (Fig. 3A). *C*_s_ is also located in a broad region of low *V*_P_ (~2 to 4 km/s) in the upper crust (*12*). However, the greater sensitivity of CSEM data to this feature and differences in regularization between the seismic tomography and EM inversion allow for a higher resolution image of *C*_s_ to be obtained in the resistivity model, which shows that *C*_s_ is a distinct, rather than pervasive, subhorizontal structure within the upper crust.

*C*_d_ is not associated with a low-velocity zone (Fig. 3B). Instead, whereas the *V*_P_ of *C*_d_ is typical for young, Pacific lower oceanic crust (*12*), its conductivity deviates markedly from values north of the OTF and oceanic crust in other regions (*19*–*21*, *24*). For context, the highest conductivities in lower oceanic crust have previously been observed in MOR partial melts and associated hydrothermal circulation (*20*, *28*), and even these values are more resistive (~30 ohm-m) than *C*_d_ (2 to 10 ohm-m).

A high-conductivity region, *C*_FZ_ (≤ 20 ohm-m), extends into the upper crust beneath the intersection of each profile with a fracture zone (FZ) north of the active Gofar OTF (Figs. 1 to 3). Although this feature occurs at the edge of our active-source transmissions, it coincides with a low-velocity zone (Fig. 3A) imaged from seismic refraction data (*12*) and thus bolsters observations that FZs are sites of enhanced porosity, and hence increased fluid content, for oceanic crust [e.g., (*29*)].

## DISCUSSION

### Nature of conductivity anomalies at Gofar OTF

To gain insight into the nature of the crustal conductors, *C*_p_, *C*_d_, and *C*_s_, we estimated the fluid volume fraction (porosity) necessary to explain these features assuming a seawater pore fluid composition (fig. S6; Materials and Methods).

#### Intense damage and deep fluid infiltration

The vertical conductor, *C*_p_, extends well into the lower crust, and our models suggest that it has an average porosity of 3%, with a maximum of 5% (fig. S6). This high porosity required for *C*_p_ is consistent with its correspondingly low *V*_P_ (*12*) and location adjacent to, and potentially within, the damage zone created by brittle deformation in the transform domain. Observations of high porosity that extends through the entire oceanic crustal section are generally rare as overburden causes pore space closure with depth. While deep penetration of seawater in OTFs has been suggested on the basis of microstructural and mineralogical analyses of peridotite mylonites of the Shaka and Garrett OTFs (*30*, *31*), and inferred from low-velocity zones in seismic tomography experiments at the Romanche and Gofar OTFs (*12*, *32*), our results present an independent line of evidence for this. Our resistivity models confirm that deep fluid infiltration, extending through the lower crust in the plane of the transform fault domain, is a feature of the barrier zone within Gofar and that hydration is at least as laterally pervasive as our profile line spacing (~8 km).

#### Lower crustal brines

Our modeling of the subhorizontal conductor, *C*_d_, in the lower crust suggests an average porosity of 9% and a maximum of 16% assuming that seawater is the pore-filling fluid (fig. S6). These values are unrealistically large for lower crustal depths, and thus seawater alone cannot be the explanation for this feature.

As an alternative hypothesis for the high conductivity of *C*_d_, we converted the conductivity of *C*_d_ to melt fraction because partial melts have been shown to explain high-conductivity anomalies for EM studies in ridge settings (*20*, *28*). Assuming a temperature of 1200°C for *C*_d_, we found that *C*_d_ requires melt fractions up to 69% (*33*) (fig. S7; Materials and Methods). This is an unrealistically large volume of melt that would create a clear low-velocity zone in the seismic tomography model of (*12*). Further, the modeled temperature structure for the Gofar lower crust is estimated to be <600°C [fig. S6; (*11*, *12*, *34*)], suggesting that partial melt should not persist. Thus, melt alone cannot explain the anomalously high conductivity of *C*_d_.

Under appropriate pressure-temperature conditions, phase separation of saline fluids will lead to the formation of a low-salinity vapor and a dense, salt-concentrated brine (*35*). In MOR settings, brines accumulate during hydrothermal circulation of seawater into the crust above an axial melt lens or deeper melt reservoir (*36*–*39*). Brines may also be generated during exsolution of cooling magma bodies, which has been invoked to explain intermediate depth (~5 km) conductivity anomalies beneath continental volcanoes (*40*). Owing to their large free-ion content [in some cases >50 weight % (wt %) NaCl], brines have conductivities of tens to hundreds of siemens per meter (10^−2^ to 10^−1^ ohm-m) (*41*), much greater than that of seawater and volatile-poor basaltic melt (*33*). Although trade-offs between the initial bulk salinity of the brine-generating fluid and the total amount of brine condensation preclude exact porosity estimates for *C*_d_, if we assume a brine of 25 wt % NaCl completely fills the pore spaces of *C*_d_, then the porosity required to explain this feature is at most ~7% and on average ~4% (fig. S8; Materials and Methods). We note that a higher brine salinity and potentially hotter temperatures of brine formation will correspond to lower required porosity, but 25 wt % NaCl and 525°C are the maximum verified salinity and temperature in the formulation of (*41*). Thus, the most feasible explanation for the high conductivity of *C*_d_ in the lower crust at Gofar is brine-filled pore spaces rather than seawater or only partial melt.

Brines have also been implicated as an explanation for the high-conductivity anomaly found asymmetrically about the Dead Sea Transform Fault, which, similarly to *C*_d_ at Gofar, is not characterized by a prominent low-velocity zone (*42*). Because seismic velocity is unaffected by ionic composition, this interpretation is consistent with both our EM data and the seismic tomography model of (*12*).

#### Upper crustal brines

We estimate an average porosity of 13% and a maximum of 30% for the subhorizontal conductor *C*_s_ in the dike section of the upper crust (fig. S6). This porosity is unreasonably large for unfaulted upper oceanic crust, which is estimated to have porosities ranging from ~1 to 10% (*18*, *19*, *23*). Yet, the subhorizontal orientation of *C*_s_ and its asymmetry about Gofar suggest that *C*_s_ was not caused by vertical fracturing related to OTF damage.

Instead, it is likely that *C*_s_ also represents brine condensation at the base of a hydrothermal cell (*38*, *43*), although it is possible that *C*_s_ simply maps a region of heightened permeability within the upper crust or is related to a remnant off-axis melt lens (*44*). Any of these interpretations would imply our seawater-filling porosity estimates are inaccurate.

### Mechanism for asymmetric brine formation at a fast-slipping OTF

The large, subhorizontal crustal conductivity anomalies, *C*_d_ and *C*_s_, are most likely explained as brines generated from hydrothermal circulation of seawater that reached pressure-temperature conditions for phase separation. The presence and asymmetric distribution of these conductors are the most curious features of our resistivity models.

If brines were ubiquitous in oceanic crust, then they should have been identified in other marine CSEM surveys, which, unlike passive-source EM data, record high-frequency signals that constrain crustal structure. Yet, CSEM data from the ultraslow spreading Mohns Ridge (*20*) and 23 to 24 Ma fast-spreading-derived Cocos Plate near the Middle America Trench (*19*) do not show evidence for subhorizontal crustal conductors, which implies that brines are not pervasive in oceanic crust.

It is possible that brines are unique to oceanic crust of young and fast-spreading derived lithosphere, but because the northern side of the Gofar OTF lacks prominent subhorizontal crustal conductors, this seems unlikely. A key difference between the lithosphere that comprises the northern and southern sides of Gofar is proximity to the main branch of the EPR. The plate north of the OTF was formed from a presumably low melt supply intratransform spreading center, whereas the plate to the south was derived from the main EPR, a source of robust magmatism only ~40 km west of our profiles. While this contrast could mean that brines are actively forming only at the EPR axis in the crust south of Gofar and not at the intratransform spreading center axis to the north, which would imply that *C*_d_ and *C*_s_ are remnants of axial processes, this is unlikely for two reasons. First, theoretical analyses on the dynamics of brine storage (*38*) do not indicate crustal residence times that reach the ages of the lithosphere at Gofar (≥0.5 Ma). In addition, numerical flow simulations of brine condensation and mobilization (*45*) imply that a permeable zone like *C*_p_, which extends through the crust and allows for deep seawater infiltration, would lead to brine dilution and upwelling in the absence of active brine formation. We thus propose that the crustal brines imaged as *C*_d_ and *C*_s_ are currently forming at Gofar, and we suggest that their formation is intimately linked to the presence of the OTF and proximity to a robust magma supply.

**Fig. 4.**
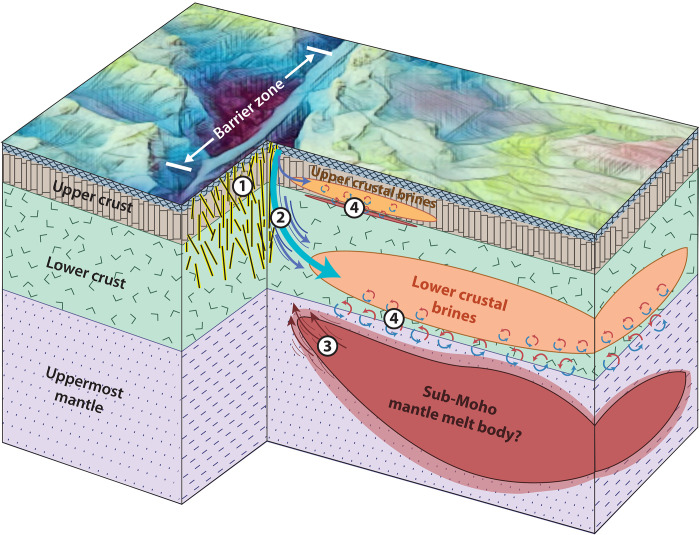
Schematic interpretation of electrical resistivity models in the context of the Gofar transform fault. Diagram illustrates the barrier zone region of the Gofar OTF and its uppermost lithospheric structure. (1) Our results indicate that there is intensified fracturing along the fault plane of the transform valley. (2) Enhanced permeability from the fracturing along with a heat source in the uppermost mantle have driven deep fluid flow. (3) Melt may migrate into the transform domain or be injected into the uppermost lithosphere from the main branch of the EPR south of Gofar, which could provide the differential heat source driving asymmetrical fluid drawdown and phase separation. Alternatively, melt may derive from localized decompression melting. (4) Hydrothermal circulation leads to phase separation and condensation of brines in the crust. Conductors *C*_s_ and *C*_d_ are shown as anomalies in the upper and lower crust, respectively. *C*_p_ is represented as the fault plane fracturing and blue fluid flow arrows.

Brine can be generated from seawater circulating around a cooling magma body, with heat from the magma driving phase separation of the saline fluids. Our resistivity models show that the OTF provides a permeable, high-porosity pathway for seawater to reach lower crustal depths (*C*_p_). The asymmetrical occurrence of the brines (*C*_d_ and *C*_s_) requires that phase separation of this seawater acts preferentially to the south of the OTF, which necessitates a differential driving mechanism that operates exclusively on the side of the fault closer to the main EPR. We propose that the presence of partial melt in the mantle near the OTF has promoted the development of crustal brines at Gofar by providing a thermal anomaly that has driven increased hydrothermal circulation south of the OTF (Fig. 4). This partial melt may be sourced from the EPR or locally through decompression melting.

Traditionally, OTFs have not been considered to host magmatic and hydrothermal processes. However, geodynamical simulations of ridge-transform systems demonstrate that conditions in melt-rich, fast-slipping environments allow for the migration and extraction of melt into the transform domain (*46*–*48*). Models also show that temperatures are elevated near the center of OTFs relative to their segment ends, which can both promote melting and the transport of off-axis melts into the OTF (*34*). These numerical models support observations of negative gravity anomalies in fast- and intermediate-slipping OTFs, inferred to indicate thickened crust from ridge-adjacent magmatic accretion (*5*). Small melt anomalies in the lower crust at distances up to 22 km off-axis have been indicated in seismic refraction and reflection experiments (*44*, *49*–*51*). In addition, Ar-Ar dating on the 8°20′N seamount chain of the EPR demonstrates the tapping of far-off-axis melt at distances as great as ~90 km from the ridge (*52*). Furthermore, the abundance of intratransform spreading centers segmenting fast- and intermediate-slipping OTFs suggests that conditions that favor melting or melt sources must be accessible far-off-axis from the spreading center at certain OTFs. By imaging the saline products of recent hydrothermal circulation in the crust south of Gofar, our resistivity results support claims that melt can influence OTFs.

While this melt may be derived from the EPR, it might be generated locally in the survey region. A transverse ridge just south of the fault valley (Fig. 1B) is potentially indicative of flexural uplift south of Gofar (*53*). Such uplift may have been sufficient to trigger localized decompression melting in the vicinity of the transverse ridge.

We posit that *C*_d_ and *C*_s_ represent the “Goldilocks case” of crustal brine formation unique to OTFs. We suggest that melt has migrated from the ridge into the mantle south of Gofar (*47*, *48*), was injected into the young lithosphere from the EPR (*44*, *54*), or has perhaps formed locally as a result of flexural uplift (*53*). The presence of such a thermal anomaly, coupled with the increased permeability of the OTF damage zone (*C*_p_), has driven deep fluid flow into the crust and led to brine formation. It is unknown how much of this melt has crystallized in place or whether isolated and seemingly undeformed circular seamounts just south of the fault valley (Fig. 1B) may represent its surface influence. We speculate that melt within transform domains may be a frequent occurrence at intermediate- and fast-slipping OTFs that helps drive hydrothermal processes far-off-axis from ridges. Such hydrothermal circulation at OTFs would have implications for abyssal life that thrives on chemosynthesis, geochemical exchange between seawater and the lithosphere, evolution of the lower crust, and the global heat flux (*39*). In addition, our findings of deep fluid infiltration at Gofar have ramifications for earthquake dynamics at OTFs and the ability of OTFs to hydrate the oceanic crust that will eventually expel this water at subduction zones.

## MATERIALS AND METHODS

### CSEM data acquisition

We collected CSEM data on 27 ocean-bottom EM receivers (OBEMs). The OBEMs are Mk III broadband receivers with 10-m-long electric dipoles and induction coil magnetometers, which can measure the orthogonal components of horizontal electric and magnetic fields (*55*). Acquiring CSEM data involved deep-towing the Scripps Undersea Electromagnetic Source Instrument (SUESI) near the seafloor to maximize coupling of the source current to the seafloor and to minimize its attenuation through seawater (*55*). We attempted to maintain an altitude of 100 m for SUESI, but we flew SUESI higher off the seafloor to avoid collision with steep topography in the deepest parts of the fault valley. SUESI output a ~300 A alternating current across a 293-m horizontal electric dipole terminated by copper electrodes. We chose the complex binary Waveform D of (*56*) as the form of the source current and used a fundamental transmission frequency of 0.25 Hz. This doubly symmetric waveform is advantageous because its power is spread among several harmonics, which enables us to characterize the resistivity structure at various length scales.

### Processing and inversion of the CSEM data

Before processing the CSEM time series, we removed data from any OBEMs that showed evidence of unusable electric field channels, which resulted from poor electrode connections or intolerably high-noise levels. This led to the omission of data from two OBEMs on profile GTF-4. In addition, we did not use the data from another OBEM on GTF-4 in our analysis because of ambiguity in its seafloor geometry that could not be adequately ascertained. The remaining 24 OBEMs recorded low-noise data of high quality (fig. S9).

We used the method described in (*56*) to robustly process the data. We first divided the time series into 4-s, nonoverlapping windows. We generated Fourier coefficients for these windowed time series by prewhitening, Fourier transforming, and postdarkening each segment. We then normalized the coefficients by the source dipole moment and corrected for the unique sensor response of each OBEM. To reduce the variance in our estimates of amplitude and phase, we stacked the Fourier coefficients into 60-s-long segments using a routine that iteratively removed outliers. Data errors were estimated on the basis of the residuals of these stacks. The minimum allowable error on each data point was set to 2% (in other words, the error floor was 2%). We removed all data with signal-to-noise ratios ≤3 and any obvious outliers that persisted after stacking.

Modeling CSEM data requires that the source geometry be well constrained. To navigate the location and orientation of SUESI in the water column, we applied the inverted long-baseline acoustic navigation method described in (*57*). While this approach is expected to provide accuracy to within 5 and 37 m in the inline and crossline positions of the towpath, respectively, the rapid decay of the electric field amplitude at short transmitter-receiver offsets can result in large errors for even such small navigational uncertainty at short offsets (*58*, *59*). For this reason, we excluded all amplitude data at offsets ≤2.5 km and all phase data at offsets ≤5 km. In addition to removing these short-offset data, we estimated the error arising from geometric uncertainties in both the transmitter and OBEM locations and orientations (*58*) and added this to the error estimated from the stack residuals. Last, because small clock errors increasingly contaminate high-frequency phase data, we removed all phase data above 1.75 Hz from our analysis.

We used a uniform, 1 ohm-m halfspace as the starting model for each inversion to prevent imposing a structural bias on the model. The root mean square (RMS) misfits of these halfspace starting models were 139.05, 166.65, and 114.61 for GTF-3, GTF-4, and GTF-5, respectively. The preferred models shown in Fig. 2 each converged to RMS misfits of 1.00 after 13, 21, and 21 iterations for GTF-3, GTF-4, and GTF-5, respectively. Modeling studies were performed to test the effect of incorporating anisotropy into the inversion. These studies suggest that anisotropy is not required to fit the data and that the isotropic models presented are suitable (see figs. S1 and S2).

### Sensitivity analyses

We performed many sensitivity studies to assess the ability of the data to constrain certain model features. In general, it is important to be aware that EM is a diffusive method and EM data are particularly useful in determining the conductance of a material, that is, its conductivity-thickness product. Because of this, and due to regularization applied in the inversion algorithm, a feature that appears in the model may, in reality, be smaller and more conductive.

#### Conductivity anomalies

To test for the presence of the conductive anomalies *C*_p_, *C*_s_, and *C*_d_, we first identified the model mesh cells within each conductor that were less than a cutoff of 5, 10, and 30 ohm-m. We then replaced the identified cells within each conductor with resistive material up to the cutoff (i.e., 5, 10, or 30 ohm-m) and computed the forward response of the resulting model. The results of these sensitivity tests are shown in tables S1 to S3 and fig. S10. Note that because no cells in *C*_p_ are less than 5 ohm-m, we only computed the forward response for the 10 and 30 ohm-m case. The tests confirm that the data are most sensitive to the shallowest conductor, *C*_s_, which is to be expected given the depth attenuation of the CSEM source. The results suggest that all conductors are required by the data but that they may be more resistive than the preferred model values. With the exception of GTF-4, prescribing the conductors *C*_s_ and *C*_d_ to be no less than 5 ohm-m had a negligible effect on the RMS misfit, implying that these conductivity anomalies may be slightly more resistive than the preferred models indicated in GTF-3 and GTF-5. In all cases, the RMS misfit increases when *C*_s_ and *C*_d_ are forced to be at least 10 ohm-m. For all conductors, the RMS misfit is significantly larger when they are forced to be no less resistive than 30 ohm-m.

To build additional confidence that these conductivity anomalies are required by the data, we re-inverted the data from a uniform, 1 ohm-m halfspace except for the model mesh cells contained in *C*_p_, *C*_s_, and *C*_d_. We imposed bounds on these model cells such that the resistivity in these cells could not be allowed to converge to less than 10, 30, and 50 ohm-m. All 27 inversion models were able to converge to RMS misfits of 0.99 to 1.01; however, each model included a film of more conductive material around or just above the bounded region (fig. S11). It seems the models compensated for the inability to insert *C*_p_, *C*_s_, and *C*_d_ in the preferred locations by making the surroundings unrealistically conductive. This again implies that the conductors are required by the data.

#### Sensitivity to a mantle conductor

A natural question that arises from our resistivity models is whether the lower crustal conductor, *C*_d_, extends to or is potentially fed by a mantle melt body. To address this question, we must first determine whether our data are sensitive to such a mantle conductor.

To test for the sensitivity of our data to a mantle conductor beneath *C*_d_, we inserted a 5 ohm-m, 5 km by 5 km conductor in the mantle at depths of 10 to 15 km beneath the *C*_d_ conductor in each profile’s preferred model (fig. S5). We calculated the forward response of these models and found that the resulting RMS misfits were each 1.01. These tests show that the mantle conductor did not significantly change the fit of the data to the models and thus suggest that our CSEM data alone cannot verify or refute the presence of a conductor in the mantle beneath *C*_d_.

#### Resistivity gradient north of fault

We observe a subtle decrease in resistivity in the lower crust north of the Gofar OTF for profiles GTF-3 and GTF-4 at depths of ~6.5 to 10.8 km and ~6.0 to 9.5 km, respectively (Fig. 2, A and B, and fig. S4A). It is unclear whether these are artifacts of the inversion or if they represent true changes in the resistivity gradient north of the fault. As a test, we re-inverted GTF-3 and GTF-4, forcing the lower crust from 2.5 and 3.2 km north of the fault, respectively, at depths ≥6.15 and ≥5.5 km, respectively, to be at least 500 ohm-m. The resulting models converged to RMS 0.99 and RMS 1.00, and the low-resistivity zone was no longer obvious in the bounded inversion (fig. S4, B and C). On the other hand, all cells surrounding the original low-resistivity zone converged to values more conductive than the preferred model (fig. S4, D and E). This is less apparent than the sensitivity tests performed for the conductors *C*_p_, *C*_s_, and *C*_d_, so we show the difference between the bounded and preferred resistivity models in fig. S4 (D and E). Given these results, it remains unclear whether the slight resistivity decrease within part of the lower crust north of the fault is real or a model artefact. Future geophysical datasets are needed to assess this further.

### Porosity calculations

To estimate porosity from our resistivity models (figs. S6 and S8), we apply the widely used empirical relationship known as Archie’s law (*60*)

ϕ=(ρfρ)1m (1)

where ϕ is the porosity of the rock, ρ is its bulk resistivity, ρ*_f_* is the resistivity of the pore-filling fluid, and *m* is the cementation exponent, a parameter that accounts for the connectivity of the pore spaces. As was demonstrated in (*59*), we will neglect surface conduction from ion mobility in clays as it is negligible when compared to that of seawater and brines. In addition, clays are not stable at temperatures greater than ~60° to 150°C (*61*). Other models exist that attempt to relate porosity and electrical resistivity (i.e., effective medium, pore network, percolation, fractal, or theories) (*62*). We choose Archie’s law because, although it was originally developed for sedimentary rocks, it has been demonstrated to approximate the porosity-resistivity relationship reasonably well in oceanic crust (*19*, *63*).

Lower values of the cementation exponent, *m*, in Archie’s law indicate more well-connected, crack-like pore spaces than larger *m* values. As *m* decreases, meaning the pore spaces are better connected, the porosity necessary to explain a given bulk resistivity also decreases. In the absence of laboratory constraints on the cementation exponent, we calculated porosity using *m* = 1.5 and *m* = 2. The lower value is appropriate for highly fractured rock of the extrusive layer and possibly for the subvertical conductor *C*_p_ because it may be associated with damage along the transform fault plane that leads to higher pore connectivity. Drill cores recovered from the dike section of oceanic crust indicate that vesicular pore spaces are present below the shallow-most extrusive layer, and thus logging measurements are typically fit with *m* = 2 (*23*, *64*, *65*). This is a common choice of cementation exponent in other applications of Archie’s law on resistivity models from seafloor CSEM data (*19*, *21*). Therefore, for crust beneath the extrusives and outside the transform fault plane conductor *C*_p_, it should be more appropriate to apply the larger value for the cementation exponent, which will still result in a conservative estimate of the porosity. Porosities quoted in the main text assume *m* = 1.5 for the extrusives and *C*_p_, and *m* = 2 elsewhere (figs. S6 and S8).

The pore-filling fluid is assumed to be an H_2_O-NaCl solution (seawater or brine). To determine its resistivity, ρ*_f_*, we used the formulation of (*41*), which states

$$\rho_{f}={\left(M\times \Lambda \right)}^{-1}\times 10^{3}$$ (2)

where *M* is the molarity of the saline solution in mol/m^3^ and Λ is a viscosity-dependent term defined as

$$\Lambda =A+B\mu^{-1}+C\mu^{-2}$$ (3)

where μ is the fluid viscosity in Pa s. Coefficients *A*, *B*, and *C* are defined in (*41*) and depend on the molality of the solution. We prescribe the molality of the solution to be equivalent to 3.5 wt % NaCl for a pore-filling fluid of seawater composition and 25 wt % NaCl for brine composition, the upper bound of the verified salinity range for Eq. 2. As noted in the main text, the brine salinity may be greater than 25 wt % NaCl, but we choose not to extrapolate Eq. 2 above its verified range. We use the ProBrine software of (*66*) to obtain the viscosity and density of the pore fluid (*35*, *67*). Because viscosity and density both depend on temperature and pressure, we estimate the temperature of the fluid using the model of (*12*), and we assume lithostatic pressure. We exclude porosity estimates above *T* = 525°C as Eq. 2 is not verified above these temperatures.

### Melt fraction of *C*_d_

To estimate a lower bound on the melt volume fraction necessary to generate anomaly *C*_d_, we assumed that any partial melt present would form fully interconnected films along grain boundaries (*68*), and hence its bulk resistivity (1*/*σ_bulk_) would be described by the Hashin-Shtrikman upper bound (HS+)

$$\sigma_{\text{bulk}}^{\text{HS}+}=\sigma_{\text{melt}}\left[1-\frac{3\phi_{\text{solid}}\left(\sigma_{\text{melt}}-\sigma_{\text{solid}}\right)}{3\sigma_{\text{melt}}-\phi_{\text{melt}}\left(\sigma_{\text{melt}}-\sigma_{\text{solid}}\right)}\right]$$ (4)

where σ_melt_ is the conductivity of the partial melt, σ_solid_ is the conductivity of the matrix rock, ϕ_melt_ is the melt volume fraction, and ϕ_solid_ is the solid volume fraction given by ϕ_solid_ = 1 − ϕ_melt_.

We estimate the melt conductivity using the formulation of (*33*)

$$\text{log}\left(\sigma_{\text{melt}}\right)=2.172-\frac{860.82-204.46w^{0.5}}{T-1146.8}$$ (5)

where *w* is the melt water content in wt % and *T* is the temperature in K. On the basis of geochemical analyses of basalt glasses dredged from Gofar, we let *w* = 0.50 wt % (*69*), which is an upper bound on the melt water content and will thus produce the most conservative estimate for the melt fraction. We take *T* = 1473 K.

Because olivine is the most conductive mineral phase in the siliciclastic crust and uppermost mantle, we approximate σ_solid_ as the conductivity of olivine using the SEO3 model of (*27*)

$$\sigma_{\text{ol}}=\left(\left[{\text{Fe}{}^{\circ}}_{\text{Mg}}\right]\mu_{\text{Fe}}+2\left[V_{\text{Mg}}^{\prime \prime }\right]\mu_{\text{Mg}}\right)\times 1.602\times 10^{-19}$$ (6)

where [${\text{Fe}{}^{\circ}}_{\text{Mg}}$] and μ_Fe_ are the concentration of small polarons and their mobility, respectively, and [$V_{\text{Mg}}^{\prime \prime }$] and μ_Mg_ are the concentration of magnesium vacancies and their mobility, respectively, in the olivine crystals. The small polaron and magnesium vacancy mobilities are temperature dependent and obey

$$\mu_{\text{Fe}}=12.2\times 10^{-6}e^{-1.05\;\text{eV}/\mathrm{kT}}$$ (7)

$$\mu_{\text{Mg}}=2.72\times 10^{-6}e^{-1.09\;\text{eV}/\mathrm{kT}}$$ (8)

where *T* is temperature in K and *k* is Boltzmann’s constant in eV/K. The corresponding concentrations depend on temperature and oxygen fugacity ($f_{O_{2}}$)

[Fe°Mg]=5.06×1024e−0.357 eV/kT+3.33×1024e−0.02 eV/kTfO216 (9)

[VMg″]=4.58×1026e−0.752 eV/kT+6.21×1030e−1.83 eV/kTfO216 (10)

We perform all calculations at *T* = 1473 K and assume the quartz-fayalite-magnetite buffer for oxygen fugacity.

Figure S7 shows the resulting melt volume fraction estimates for the lower crustal conductor, *C*_d_, in each profile assuming that partial melt accounts for the entirety of the conductivity anomaly. Modeled resistivities require melt volume fractions up to 69% to explain *C*_d_ by melt alone, which is unrealistically high.
