# Supplementary material for: Evidence for crustal brines and deep fluid infiltration in an oceanic transform fault
Source: Sci Adv. 2025 Apr 11;11(15):eadu3661. doi: 10.1126/sciadv.adu3661 (PMC11988451; doi:10.1126/sciadv.adu3661)
Supplement: Supplementary file 1 — Figs. S1 to S11 Tables S1 to S3 [file sciadv.adu3661_sm.pdf]

Science Advances
AAAS

Supplementary Materials for

Evidence for crustal brines and deep fluid infiltration in an oceanic transform fault

Christine Chesley *et al.*

Corresponding author: Christine Chesley, christine.chesley@whoi.edu

Sci. Adv. **11**, eadu3661 (2025)
DOI: 10.1126/sciadv.adu3661

This PDF file includes:

Figs. S1 to S11
Tables S1 to S3

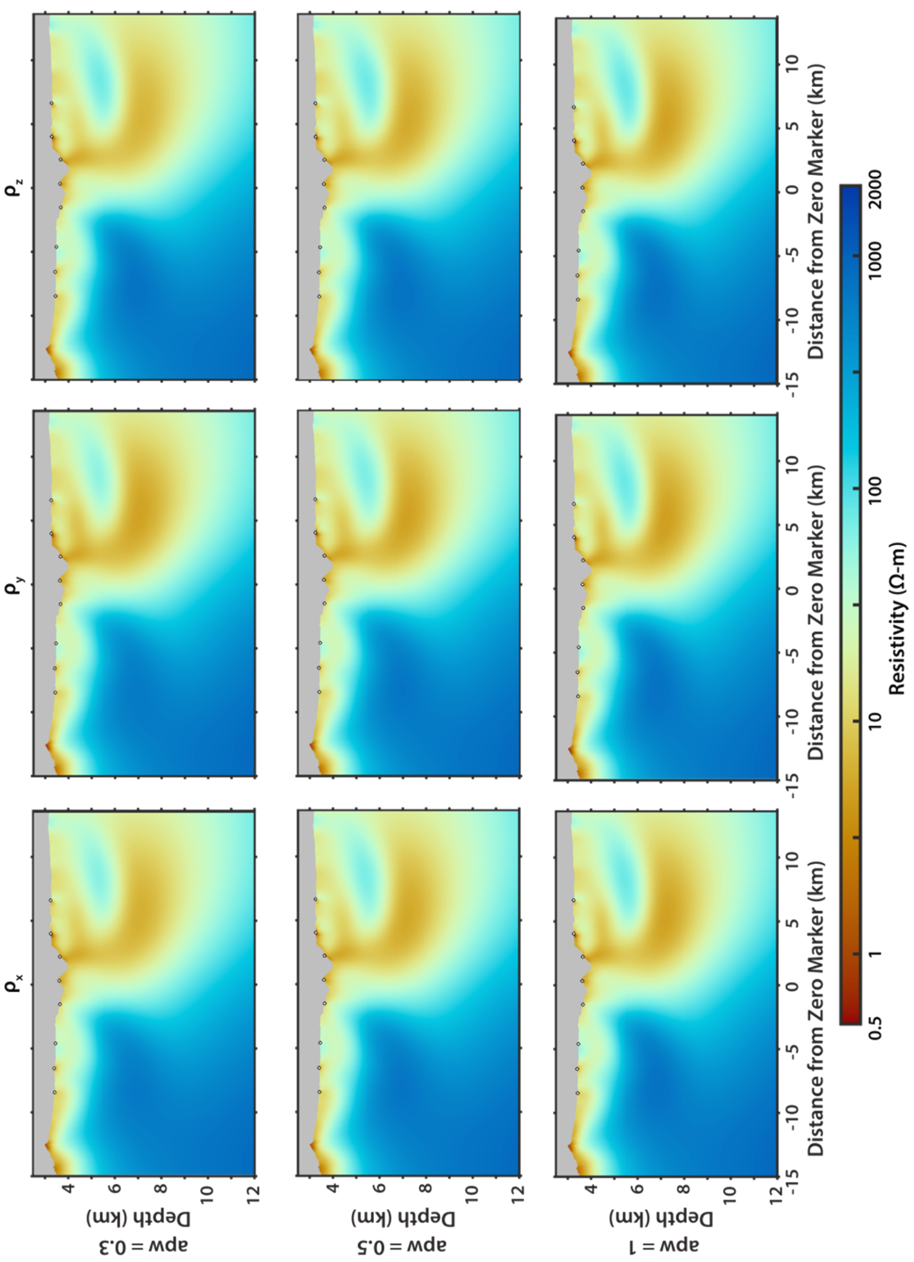

Fig. S1

Triaxially anisotropic inversion models of GTF-3. Anisotropy in the along profile (ρ_x), profile perpendicular (ρ_y), and vertical (ρ_z) directions are shown in the left, middle, and right columns, respectively. Each row shows the inversion result using a selection of different anisotropic penalty weights (apw) (23). Lowering the anisotropic penalty weight will generally produce models that are allowed to include more anisotropy. The similarity of the models for each orthogonal direction suggests that anisotropy is not required to fit the data.

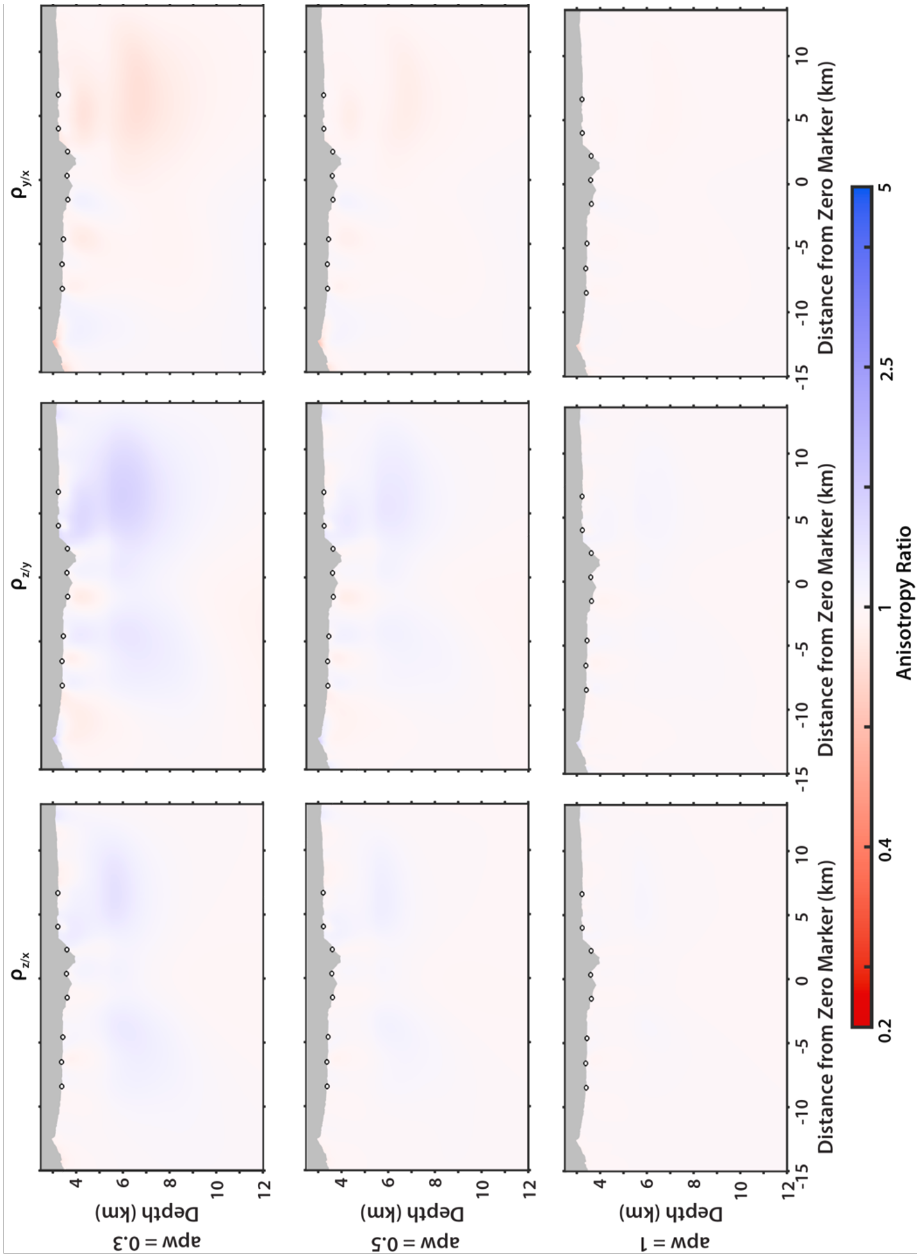

Fig. S2.

Anisotropy ratios for triaxially anisotropic inversion models of GTF-3. The ratios of the anisotropic models shown in Fig. S1 are given. The directions are as follows: ρ_x = along profile, ρ_y = profile perpendicular, and ρ_z = vertical. Each row shows the ratios resulting from a selection of different anisotropic penalty weights (apw) (23). Lowering the anisotropic penalty weight will generally produce models that are allowed to include more anisotropy, which is why the anisotropy ratios deviate from unity in the upper row. Near-unity anisotropy ratios suggest that anisotropy is not required to fit the data.

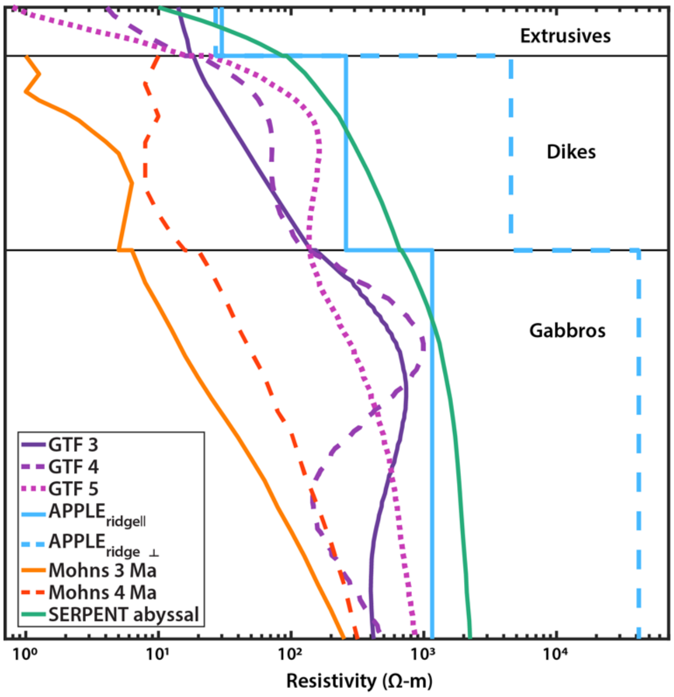

Fig. S3.

Comparison of representative 1-D resistivity values of oceanic crust from marine CSEM experiments. 1-D resistivity profiles from refs (19), (26), (20), and this study were scaled by typical thicknesses of the extrusive, dike, and gabbro layer for comparison. We assumed the sediment layer at Gofar is negligible, and ref. (20) attest that the Mohns Ridge profiles start from the dike layer. Dark purple solid, light purple dashed, and magenta dotted lines are average resistivity values from -9 km to -6 km at GTF-3, GTF-4, and GTF-5, respectively. Anisotropic model of 33 Ma Pacific lithosphere from ref. (26) in the paleo-ridge parallel and paleo-spreading parallel directions are shown as light blue solid and dashed lines, respectively. Profiles from the Mohns Ridge at 3 Ma and 4 Ma from (20) are shown as orange and dark orange solid and dashed lines, respectively. Green line is average resistivity from the Cocos Plate abyssal plain near the Middle America Trench from ref. (19).

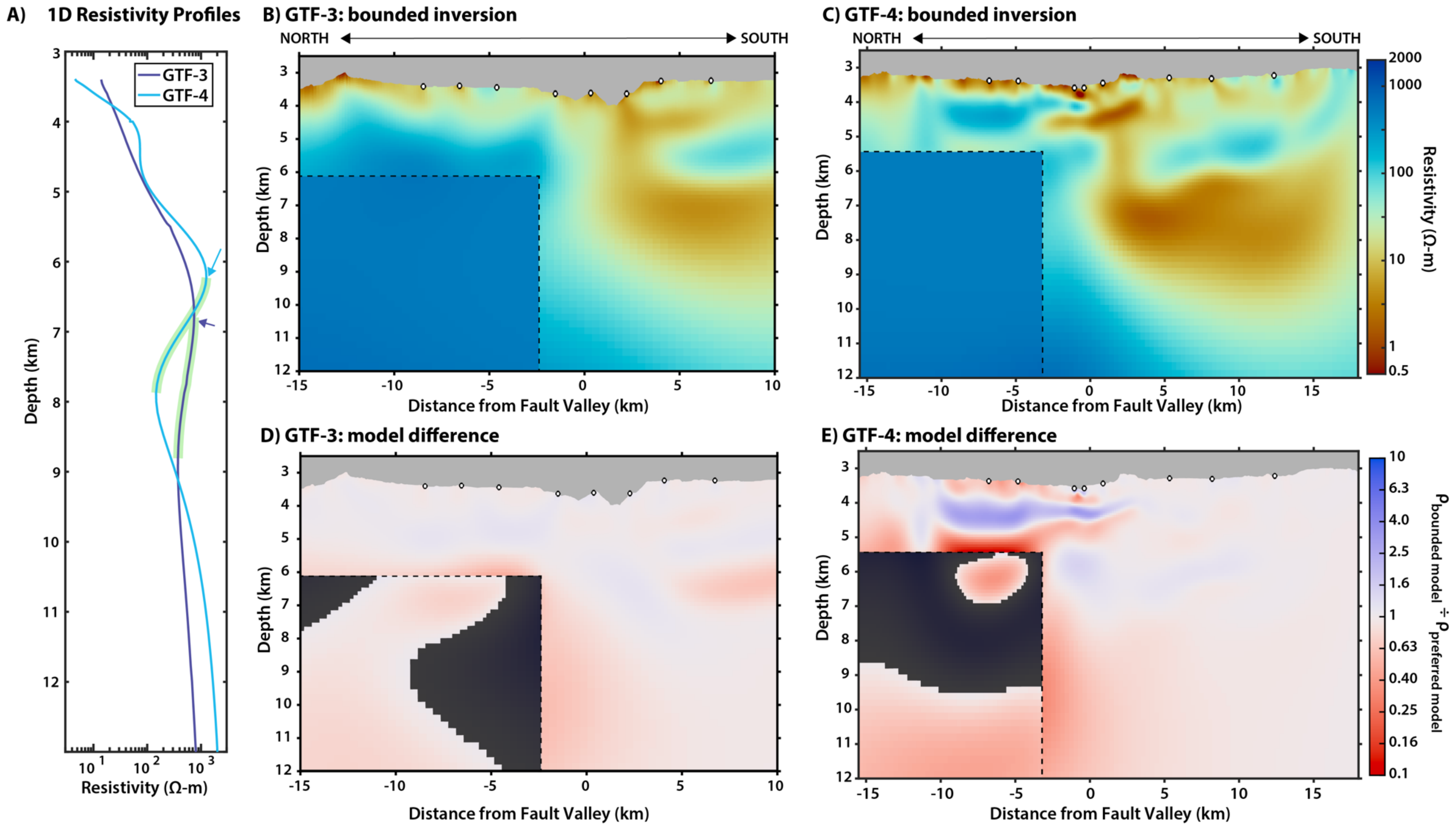
A) 1D Resistivity Profiles
GTF-3
GTF-4
Depth (km)
Resistivity (Ω-m)
B) GTF-3: bounded inversion
NORTH
SOUTH
Depth (km)
Distance from Fault Valley (km)
C) GTF-4: bounded inversion
NORTH
SOUTH
Depth (km)
Distance from Fault Valley (km)
Resistivity (Ω-m)
2000
1000
100
10
1
0.5
D) GTF-3: model difference
Depth (km)
Distance from Fault Valley (km)
E) GTF-4: model difference
Depth (km)
Distance from Fault Valley (km)
$\rho_{bounded\ model} \div \rho_{preferred\ model}$
10
6.3
4.0
2.5
1.6
1
0.63
0.40
0.25
0.16
0.1

Fig. S4.

Sensitivity test for change in lower crustal resistivity gradient north of Gofar OTF. (A) 1-D resistivity profiles taken 7 km north of the fault in the preferred models of GTF-3 (dark blue) and GTF-4 (light blue). The light green shading highlights the depths in the lower crust where the resistivity decreases with depth, thus causing a subtle anomaly north of the fault. Arrows point to the top of the change in the resistivity gradient. **(B)** and **(C)** are results from the bounded inversion of GTF-3 and GTF-4, respectively. The initial model for each was a 1 Ω-m halfspace except for the model cells within the dashed box, which were bounded to be no less than 500 Ω-m. **(D)** and **(E)** display the difference between the bounded model resistivity and the preferred model resistivity for GTF-3 and GTF-4, respectively. The color scale gives the log-spaced resistivity difference defined as the resistivity from the bounded model divided by the resistivity of the preferred model in Fig. 2B & C. Blue colors show where the bounded model is more resistive than the preferred model; reds indicate where the bounded model is more conductive. The shaded region of the box indicates where the preferred model was ≤ 500 Ω-m. Notice that the cells surrounding this shaded region are more conductive than the original model.

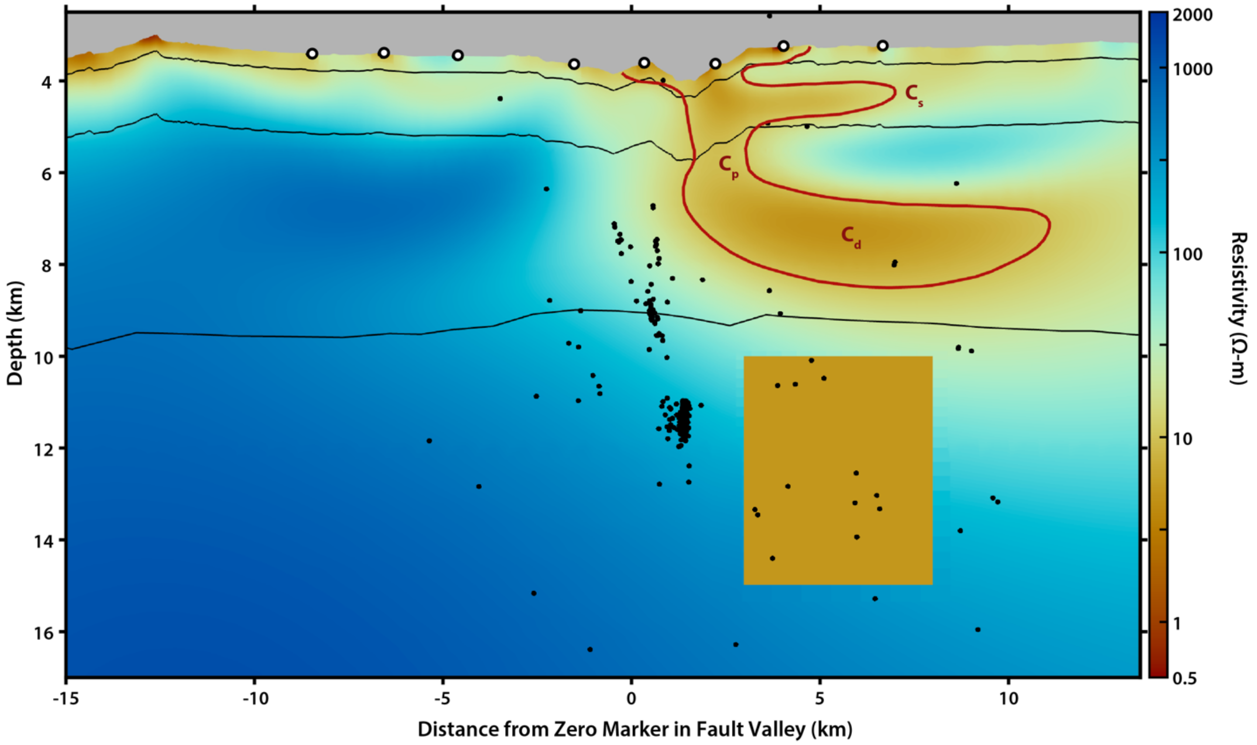

Fig. S5.

Example model setup for sensitivity analysis (forward response) of a 5 Ω-m mantle conductor beneath C_d. A 5 km x 5 km, 5 Ω-m conductor was placed in the mantle beneath C_d for each of the preferred models shown in Fig. 1, and the forward response was calculated. We show here the setup for GTF-3, although the setup is identical for GTF-4 and GTF-5. In each case, the RMS misfit of the forward response was 1.01, which implies that we cannot determine whether a conductor exists in the mantle below C_d.

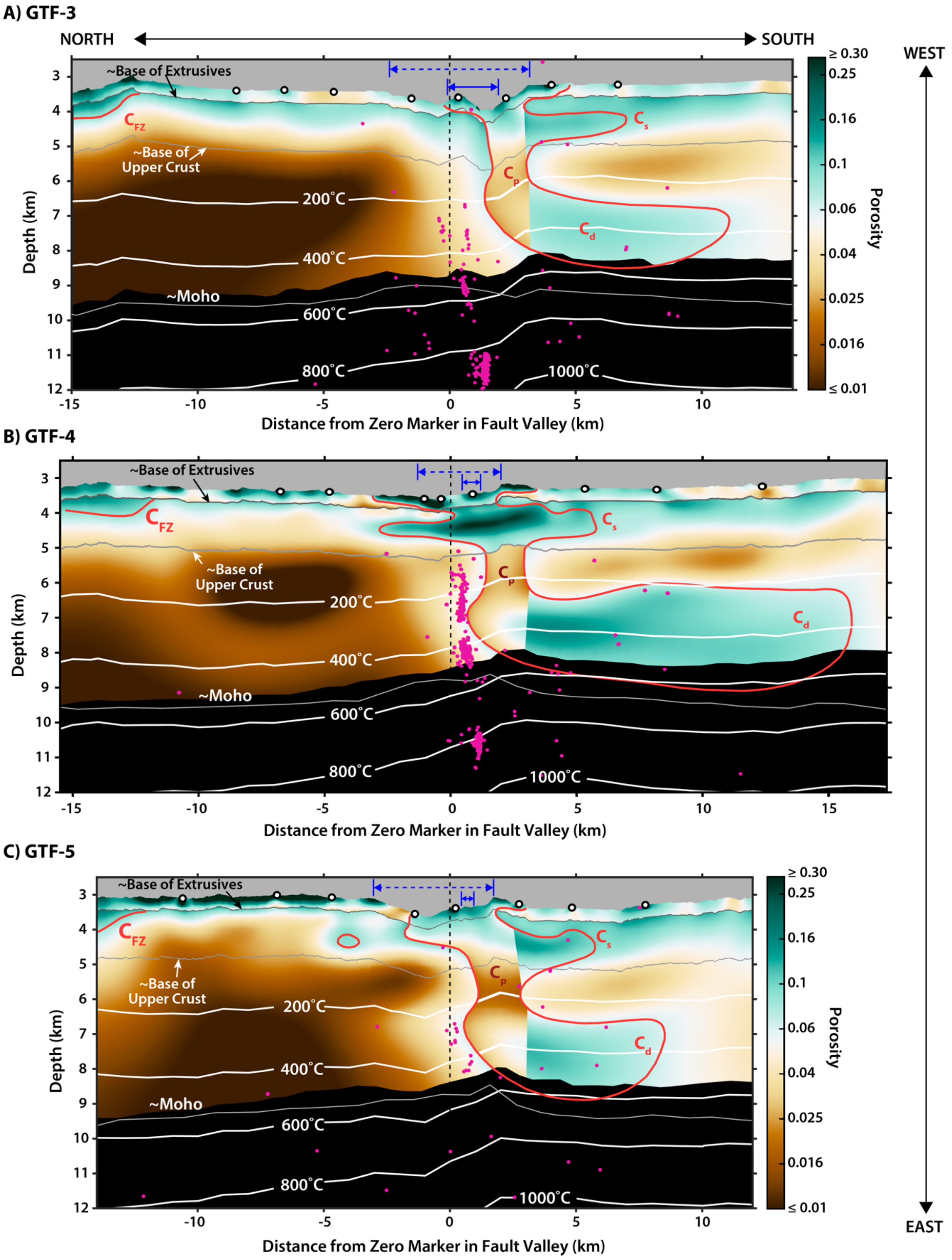

Fig. S6.

Resistivity-derived porosity assuming seawater-filling pore fluid. (A), **(B)**, and **(C)** give the porosity estimated using Archie's law with seawater as the pore fluid and the resistivity models

from profiles GTF-3, GTF-4, and GTF-5, respectively. A cementation exponent of $m = 1.5$ was used for the extrusive layer and for C_p whereas $m = 2$ was used for the remaining model domain (Materials and Methods). Isotherms are based on ref. (12) . The conductivity of seawater is estimated using ref. (43), which is verified for $T \leq 525^{\circ}$C; $T > 525^{\circ}$C are blacked out. Conductivity anomalies C_p, C_s, C_d, and C_{FZ}, which are discussed in the text, are outlined in red. Seismicity in Fig. 1 (10) within 250 m of each profile is shown as magenta dots. Approximate layer boundaries are shown as gray lines where the base of the extrusives (i.e., Layer 2A) and base of the upper crust were estimated by averaging the depth below seafloor to the 20 Ω-m and 200 Ω-m contours, respectively, from -9 to -6 km along each profile. The Moho boundary is approximated from ref. (12) by adding its depth below seafloor to each profile's topography.

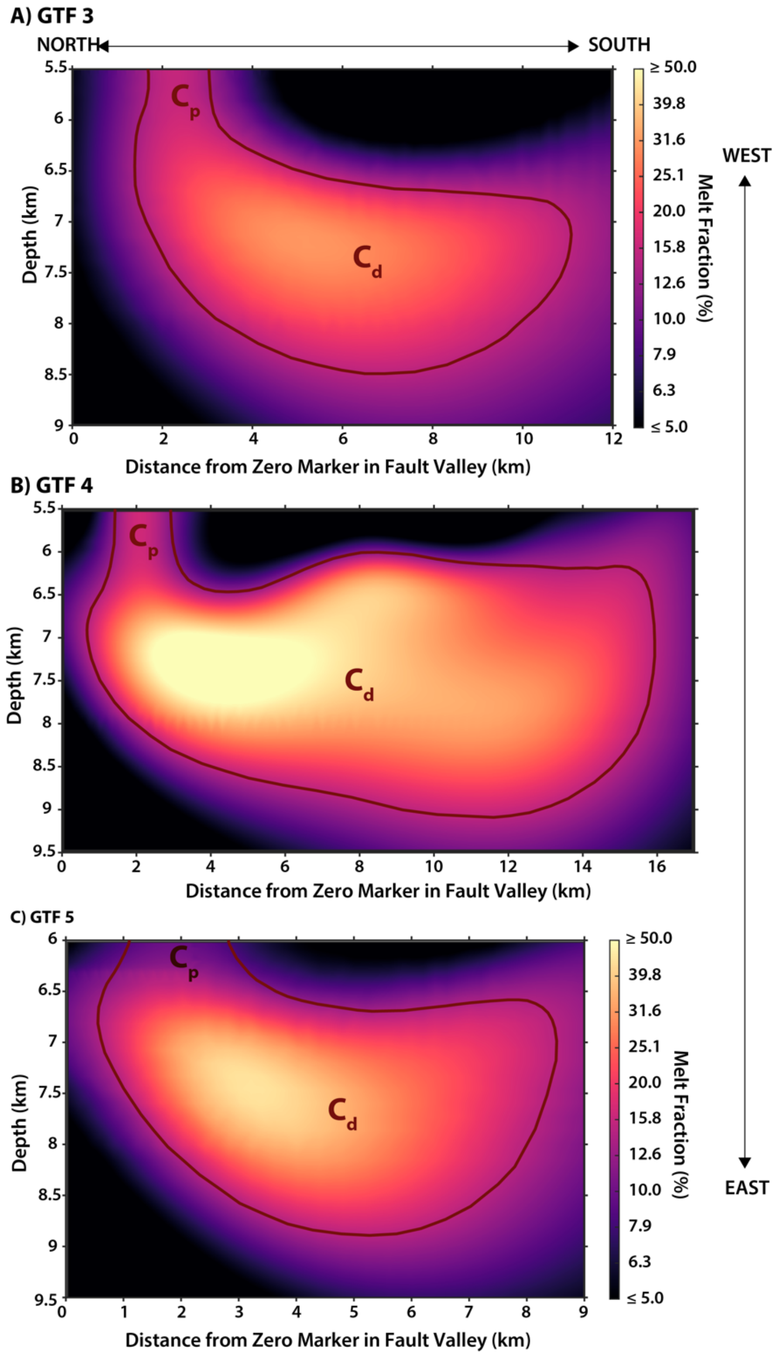

Fig. S7.

Melt fraction estimates from resistivity models. (A), **(B)**, and **(C)** give the melt fraction of C_d for GTF-3, GTF-4, and GTF-5, respectively, estimated using the formulation of ref. (35) and assuming a temperature of $T = 1200^{\circ}C$ (1473 K). The calculated melt fractions are unrealistically large, and so C_d cannot be explained by melt alone.

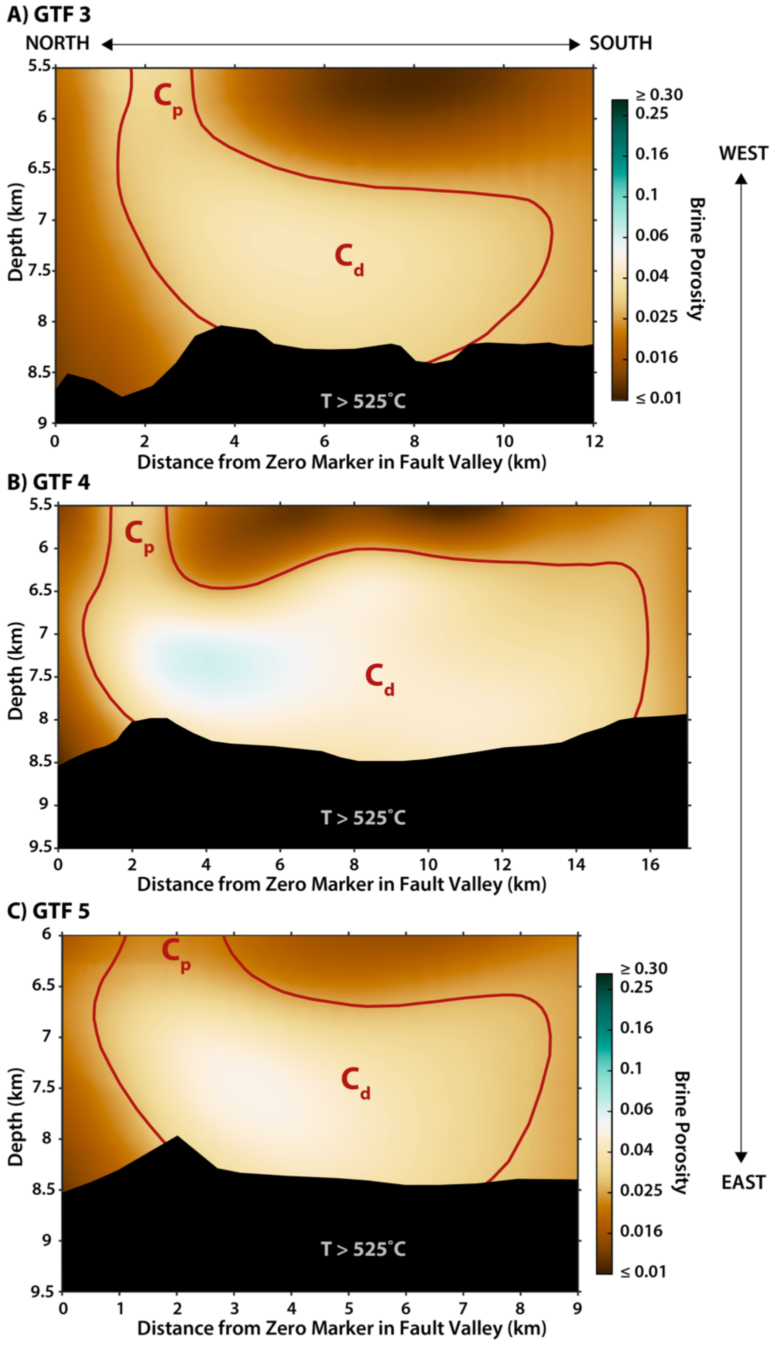

Fig. S8.

Resistivity-derived porosity for C_d assuming a 25 wt% NaCl brine solution is the pore fluid. (A), **(B)**, and **(C)** give the porosity of C_d estimated using Archie's law with brine as the pore fluid and the resistivity models from profiles GTF-3, GTF-4, and GTF-5, respectively. A cementation exponent of $m = 2$ was applied and the conductivity of the brine was calculated using ref. (43), which is only verified for $T \leq 525^{\circ}$C (Materials and Methods); $T > 525^{\circ}$C are blacked out.

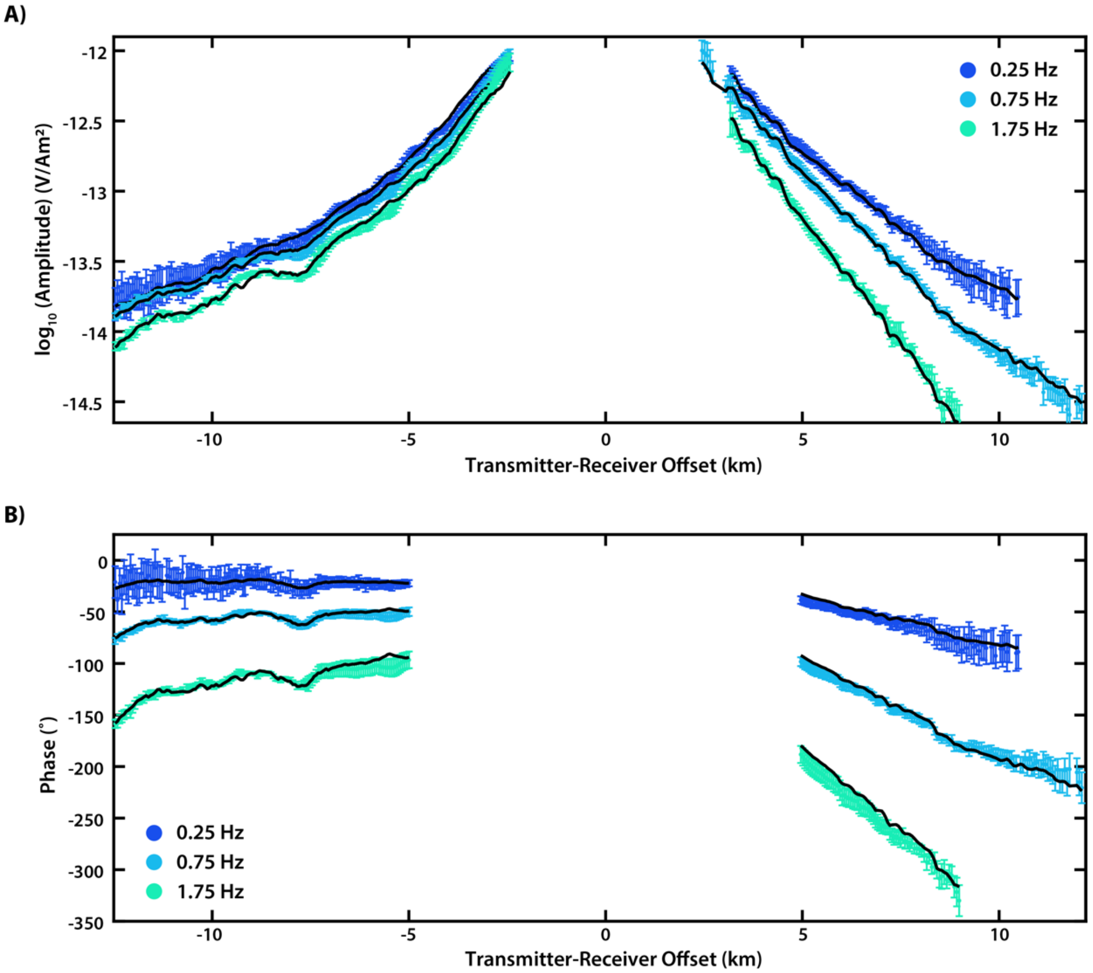

Fig. S9.

CSEM data example. (A) Amplitude and **(B)** phase data from a receiver in profile GTF-5 exemplifying the high-quality data we obtained in this survey. The data have been stacked in 60 s windows and the three highest power harmonics are shown—0.25 Hz, 0.75 Hz, and 1.75 Hz in dark blue, light blue, and light green, respectively. Error bars are the combined stacking and geometrical uncertainties. The solid black line is the model response of Fig. 2C. Note the rapid decay of phase and amplitude to the south of the fault (positive transmitter-receiver offsets). This is a clear indication that more conductive material is present to the south of the fault than to the north.

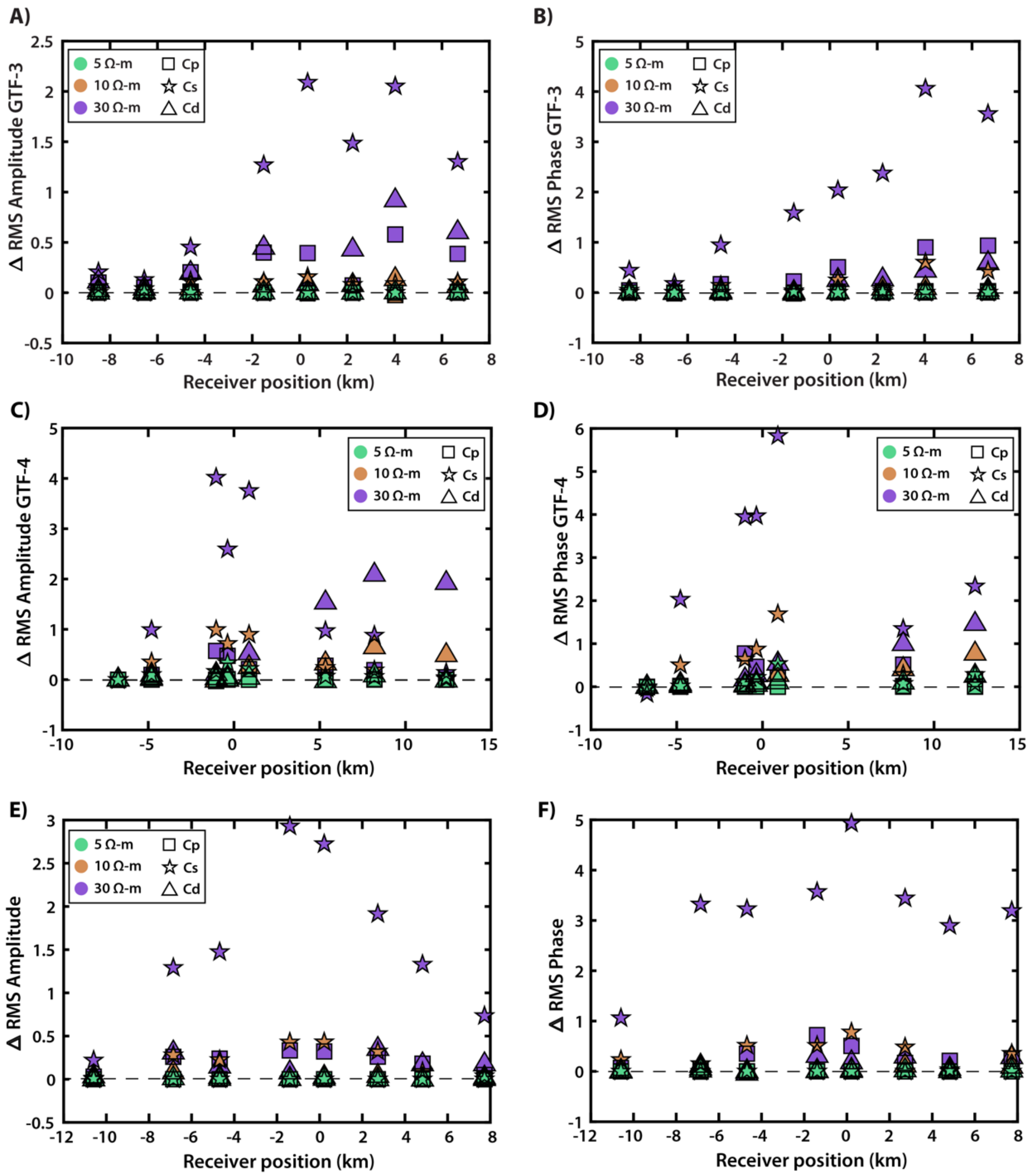

Fig. S10.

Breakdown of changes in RMS misfit from forward response sensitivity tests on presence of conductors. Each panel shows the increase in RMS misfit of a forward response compared to the preferred model for each receiver in GTF-3 **(A—B)**, GTF-4 **(C—D)**, and GTF-5 **(E—F)** of sensitivity tests for the presence of the conductivity anomalies C_p, C_s, and C_d as described in Materials and Methods. The first column are changes in the RMS misfit of amplitude data and the second column are RMS misfit changes for phase data. Square, star, and triangle symbols indicate that conductor C_p, C_s, or C_d, respectively, was analysed in the particular test. Green, orange, and purple colors indicate that the conductor was made to be no less than 5 Ω-m, 10 Ω-m, or 30 Ω-m, respectively.

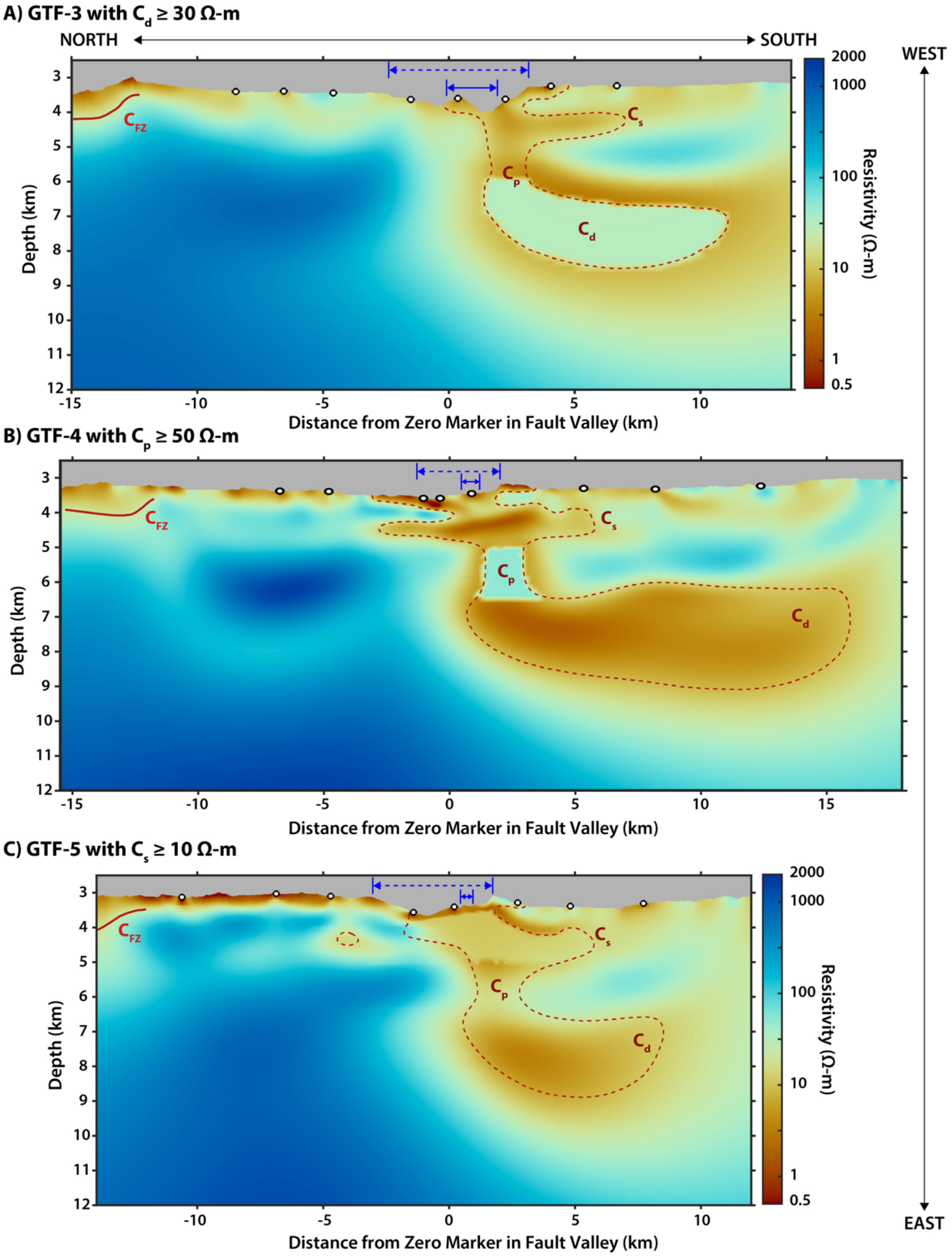

Fig. S11.

Examples of sensitivity analyses (inversions) for the presence of conductive anomalies south of the Gofar OTF. Each model shown began from a 1 Ω-m halfspace except that the model mesh cells contained in either **(A)** C_d, **(B)** C_p, or **(C)** C_s were forced to converge to no less than **(A)** 30 Ω-m, **(B)** 50 Ω-m, or **(C)** 10 Ω-m. In each case, the final model fits to an RMS misfit of 1.00, but note that each respective bounded conductor has a halo of more conductive material around it. These tests suggest that the conductors are required by the data.

	C_p	C_s	C_d
5 Ω-m	N/A	1.00	1.00
10 Ω-m	1.00	1.17	1.06
30 Ω-m	1.41	3.38	1.41

Table S1.

RMS misfit table for the forward response sensitivity tests on the presence of the GTF-3 conductive anomalies. The preferred model cells in C_p, C_s, or C_d of GTF-3 were forced to be 5, 10, or 30 Ω-m and the resulting forward response was calculated. The RMS misfit of each response is shown in the table. See description in Materials and Methods.

	C_p	C_s	C_d
5 Ω-m	N/A	1.21	1.03
10 Ω-m	1.00	1.82	1.26
30 Ω-m	1.44	4.88	2.03

Table S2.

RMS misfit table for the forward response sensitivity tests on the presence of the GTF-4 conductive anomalies. The preferred model cells in C_p, C_s, or C_d of GTF-4 were forced to be 5, 10, or 30 Ω-m and the resulting forward response was calculated. The RMS misfit of each response is shown in the table. See description in Materials and Methods.

	C_p	C_s	C_d
5 Ω-m	N/A	1.00	1.00
10 Ω-m	1.00	1.45	1.02
30 Ω-m	1.37	4.66	1.18

Table S3.

RMS misfit table for the forward response sensitivity tests on the presence of the GTF-5 conductive anomalies. The preferred model cells in C_p, C_s, or C_d of GTF-5 were forced to be 5, 10, or 30 Ω-m and the resulting forward response was calculated. The RMS misfit of each response is shown in the table. See description in Materials and Methods.
